# Intricacies of TGF-β signaling in Treg and Th17 cell biology

**DOI:** 10.1038/s41423-023-01036-7

**Published:** 2023-05-23

**Authors:** Junying Wang, Xingqi Zhao, Yisong Y. Wan

**Affiliations:** 1 Lineberger Comprehensive Cancer Center, University of North Carolina at Chapel Hill, Chapel Hill, NC 27599 USA; 2 Department of Microbiology and Immunology, University of North Carolina at Chapel Hill, Chapel Hill, NC 27599 USA

**Keywords:** TGF-beta, regulatory T cell, Th17 cell, Treg, Cellular immunity, Immunology

## Abstract

Balanced immunity is pivotal for health and homeostasis. CD4^+^ helper T (Th) cells are central to the balance between immune tolerance and immune rejection. Th cells adopt distinct functions to maintain tolerance and clear pathogens. Dysregulation of Th cell function often leads to maladies, including autoimmunity, inflammatory disease, cancer, and infection. Regulatory T (Treg) and Th17 cells are critical Th cell types involved in immune tolerance, homeostasis, pathogenicity, and pathogen clearance. It is therefore critical to understand how Treg and Th17 cells are regulated in health and disease. Cytokines are instrumental in directing Treg and Th17 cell function. The evolutionarily conserved TGF-β (transforming growth factor-β) cytokine superfamily is of particular interest because it is central to the biology of both Treg cells that are predominantly immunosuppressive and Th17 cells that can be proinflammatory, pathogenic, and immune regulatory. How TGF-β superfamily members and their intricate signaling pathways regulate Treg and Th17 cell function is a question that has been intensely investigated for two decades. Here, we introduce the fundamental biology of TGF-β superfamily signaling, Treg cells, and Th17 cells and discuss in detail how the TGF-β superfamily contributes to Treg and Th17 cell biology through complex yet ordered and cooperative signaling networks.

## Introduction

Our immune system, consisting of the innate and adaptive arms, has evolved to achieve two principal goals to maintain health. One is to recognize and tolerate entities that are deemed innocuous, or “self”. The other is to recognize and reject entities that are deemed nocuous or “nonself”. T cells are fundamental to achieve these principal goals because of T cells’ ability to recognize, with high specificity, nearly infinite numbers of antigens derived from an entity, regardless of “self” or “nonself”. For proper immunity, T cells must also properly distinguish “self” and “nonself”. Thymic negative selection eliminates T cells reacting strongly to “self”. Innate immunity enables a productive T-cell response against “nonself”. In addition, cytokines play crucial roles in balancing tolerance and immunity following the productive T-cell response. TGF-β has been recognized as a chief cytokine of “Yin-Yang” function, as it is critical for both tolerance and immunity. TGF-β was initially regarded as an immune regulatory cytokine because it suppresses proinflammatory cytotoxic and Th cells and promotes immunosuppressive Treg cells. TGF-β was later found to induce Th17 cells of both immune regulatory and pathogenic functions. It is now clear that TGF-β regulates T-cell-mediated tolerance and immunity through both Treg and Th17 cells in a context-dependent manner. Emerging evidence suggests a broad function for the TGF-β superfamily in Treg and Th17 cell biology. TGF-β superfamily signaling pathways crosstalk and interact with a myriad of other factors and pathways through multilayered mechanisms to translate complex environmental cues into defined and precise responses. Such a property of the TGF-β superfamily enables intricate control of Treg and Th17 cell function in a context-dependent manner, highlighting TGF-β’s fundamental role in immune balance. This article aims to familiarize readers with the current understanding of the TGF-β superfamily signaling and the biology of Treg and Th17 cells and to further discuss the involvement of the TGF-β superfamily in Treg and Th17 cell biology.

## TGF-β superfamily signaling is regulated at multiple molecular levels

The TGF-β superfamily consists of over 35 members, including TGF-βs, Activins, BMPs (bone morphogenetic proteins), Nodal, and GDFs (growth differentiation factors). These secreted proteins play pleiotropic roles in controlling the development, homeostasis, proliferation, differentiation, and functions of diverse cell types in health and disease [1–7]. In the immune system, TGF-β is the best studied. Activin [8–11] and BMP [12–17] also contribute to immune regulation. TGF-β has three homologs: TGF-β1, TGF-β2, and TGF-β3. While the biochemical properties of TGF-β1, TGF-β2, and TGF-β3 are similar, their expression pattern and function are tissue- and cell-type specific. Germline deletion of TGF-β2 and TGF-β3 leads to embryo lethality [18, 19]. In contrast, while TGF-β1 deletion perturbs endothelial differentiation and yolk sac hematopoiesis to a certain extent [20], TGF-β1 knockout mice can be born but succumb to severe multifocal inflammation shortly after birth [21–23]. Therefore, the principal function of TGF-β1 is to control hematopoiesis and immune function, which agrees with its preferential expression in immune cells when compared to other isoforms [24]. TGF-β1 is produced by and regulates the function of both innate immune cells, including macrophages [25–27] and dendritic cells (DCs) [28–30], and adaptive immune cells, including T and B cells [31, 32]. To control the immune responses, TGF-β1 mainly targets T cells because T-cell-specific deletion of TGF-β receptors results in a systemic, multifocal, and lethal inflammatory disease resembling the phenotypes of TGF-β1-deficient mice [33–35].

### TGF-β activation via complex posttranslational mechanisms

**Fig. 1:**
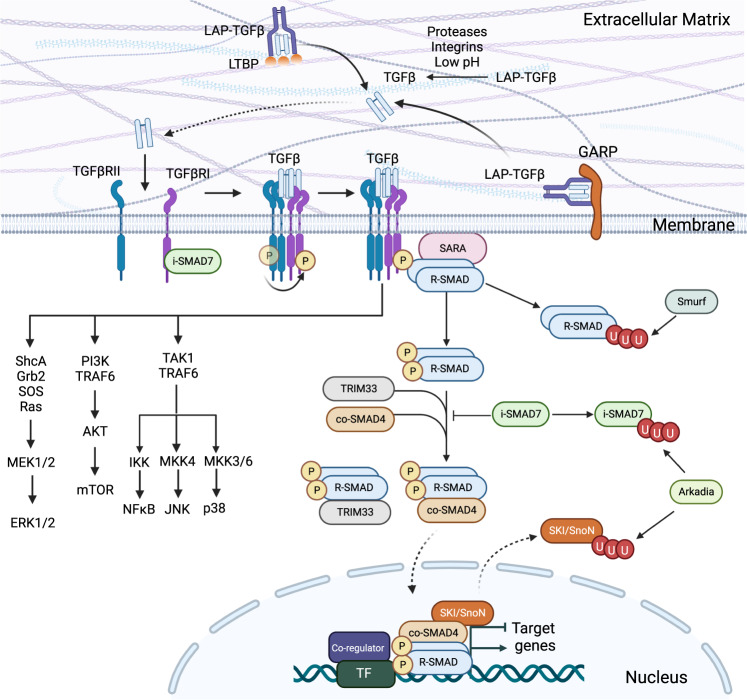
TGF-β activation and signaling. Inactive LAP-TGF-β is produced and associates with LTBP in the extracellular matrix or with GARP on the cell membrane. Active TGF-β is released from LAP-TGF-β by proteases, integrins and low pH. Activated TGF-β binds to its receptor to activate r-Smad proteins. Activated r-Smad proteins interact with co-Smad4 to translocate into the nucleus to control target gene expression by interacting with various transcription factors (TFs) and coregulators. i-Smad and SKI/SnoN proteins negatively regulate TGFβR and Smad function. E3-ubiquitin ligases, including Smurf and Arkadia, target protein degradation to modulate TGF-β signaling. TGF-β binding to its receptor also activates MAP kinase pathways, including Ras-ERK, PI3K-AKT-mTOR, and TAK1, to program cellular responses independent of Smad pathways

While the transcriptional and translational regulation of the *Tgfb* gene is poorly understood, the generation of biologically active TGF-β at the posttranslational level has been studied extensively [36, 37]. TGF-β is synthesized as a precursor molecule composed of a signal peptide, a pro-domain (latency-associated-peptide, LAP), and the mature polypeptide (TGF-β) [36, 37]. After signal peptide removal, latent LAP-TGF-β dimerizes and forms a large latent complex by binding covalently to LTBP (large-latent-TGF-β-binding-protein) through disulfide bonds to be deposited in the extracellular matrix [36]. Latent LAP-TGF-β can also disulfide-link with membrane-bound GARP (glycoprotein-A repetitions predominant protein), a protein expressed on the surface of Treg cells and platelets to position TGF-β at the cell membrane [38]. Therefore, TGF-β secretion and function tend to be localized. Unlike TGF-β, Activins and some BMPs are not secreted as a latent complex. Mature TGF-β only becomes active to induce signal transduction after being freed from LAP by proteolytic cleavage via various integrins and proteases and extracellular matrix proteins in cell-type- and context-dependent manners [39] (Fig. 1).

### TGF-β signaling via specific receptors and Smad proteins

Active TGF-β binds to its specific receptor on target cells to signal and program cellular function (Fig. 1). The signaling mechanisms of the TGF-β superfamily are conserved in both immune and nonimmune cells [40–42]. TGF-β receptor (TGFβR) is a transmembrane heterotetrametric complex consisting of two copies of ligand-specific receptor I and receptor II. Upon TGF-β binding, TGFβRII phosphorylates TGFβRI (also known as ALK (activin receptor-like kinase)-5) and activates ALK-5’s serine/threonine domain. Activated ALK-5 phosphorylates and activates receptor-associated (r)-Smad (suppressor of mothers against decapentaplegic) proteins, including r-Smad2 and r-Smad3, and promotes their disassociation from SARA (Smad anchor for receptor) [43, 44]. Activated r-Smad proteins can then translocate to the nucleus with or without associating with co-Smad4, a common Smad protein that can bind to r-Smad proteins activated by TGF-βs, Activins, and BMPs [42]. Smad-containing complexes, which often include other transcription factors and epigenetic regulators, bind to target loci in the genome to regulate gene expression positively or negatively. In addition to co-Smad4, r-Smad2 and r-Smad3 can also bind to a nuclear protein called TIF1γ (transcriptional intermediary factor 1γ), also known as TRIM33 (tripartite motif-containing 33), to control target gene expression [45]. Like TGF-β, Activins and BMPs bind to and activate their specific receptors and r-Smads to control target gene expression. The major type I receptor for Activin is ALK-4, which activates r-Smad2 and r-Smad3. The major BMP type I receptors are ALK-1, ALK-2, ALK-3, and ALK-6, which activate r-Smad1, r-Smad5, and r-Smad8. In addition to binding to ALK-5, TGF-β can bind to ALK1 or ALK2 to stimulate epithelial cell proliferation and migration [46] and to activate r-Smad1 and r-Smad5 during epithelial-to-mesenchymal transition [47]. Therefore, TGF-β can crosstalk with other members of the same family, especially Activins and BMPs, in a context-dependent manner, which can be of importance for immune regulation [8–17]. However, not all Smad proteins promote TGF-β signaling; inhibitory (i) Smad6 (i-Smad6) and i-Smad7 dampen TGF-β signaling by associating with type I receptors to prevent r-Smad activation or to disrupt the association between r-Smad and co-Smad proteins [7, 42, 48].

As the major transducers of the TGF-β signaling pathway, Smad proteins have two highly conserved MH (mad homology) domains. N-terminal MH1 and C-terminal MH2 mediate nuclear localization and protein‒protein interactions, respectively. Therefore, the MH1 and MH2 domains are important for Smads to bind to DNA and other proteins. The binding of Smads to various genetic loci and proteins enables TGF-β signaling pathways to crosstalk with a plethora of other signaling pathways to control diverse cellular functions [42, 48]. r-Smad3 and the co-Smad4 complex interact with c-Jun and c-Fos at the AP1 binding site to establish crosstalk between the pathways of TGF-β and JNK (c-Jun N-terminal kinase), a MAPK (mitogen activated protein kinase) [49]. r-Smads and co-Smad4 cooperate with LEF-1 (lymphoid enhancer-binding factor 1) and β-catenin in Wnt signaling [50]. In addition, TGF-β signaling regulates the transcription of HES-1 (hairy and enhancer of split-1) of the Notch pathway through the direct binding of r-Smad3 to NICD (notch intracellular domain) [51]. TGF-β signaling also converges with Hedgehog signaling through the co-Smad4 and GLI (glioma-associated oncogene) interaction, involving r-Smad2 and histone acetyltransferase PCAF (p300/CREB-binding protein-associated factor), to activate target genes [52]. Since the MAPK, Wnt, Notch, and Hedgehog pathways are important for T-cell functions, it is plausible that TGF-β exerts broad effects on T cells by corroborating these pathways, which warrants further study. Smad function can be regulated through posttranslational mechanisms [53–55]. For example, the MH2 domain of r-Smad can be dephosphorylated by the protein phosphatase PPM1A (protein phosphatase, Mg^2+^/Mn^2+^ dependent, 1 A)/PP2Cα (protein phosphatase-2Cα) [56] and can be phosphorylated by casein kinase Iγ2 [57] to promote r-Smad degradation. R-Smad’s linker region can be phosphorylated by p38 MAPK and ROCK (Rho-associated coiled coil kinase) [58], JNK [59], and casein kinase Iϵ [60] to positively or negatively regulate TGF-β responses. In addition, mono- and polyubiquitylation, sumoylation, acetylation, methylation, and polyadenosine diphosphate (ADP)-ribosylation of Smads regulate Smad function [53]. Notably, r-Smads, co-Smad4, i-Smads, and the activated Smad complex can be ubiquitinated by various E3 ubiquitin ligases. The best-known E3 ubiquitin ligases that regulate TGF-β signaling pathways belong to the HECT (homologous to E6AP C-terminus) family and Smurf (Smad ubiquitin regulatory factors) family, including Smurf1 and Smurf2. These E3 ubiquitin ligases target TGF-β-activated r-Smad2 and r-Smad3 and BMP-activated r-Smad1 and r-Smad5 [61, 62]. r-Smad3 is also targeted by other ubiquitin E3 ligases, including SCF (Skp1-Cullin-F-box) proteins and U-box CHIP (carboxyl terminus of Hsc70-interacting protein) [63–65]. Nuclear RNF (ring finger protein) 111, also known as Arkadia, is an E3 ligase that mediates the degradation of i-Smad7, SnoN, and SKI, which can lead to enhanced TGF-β signaling [66, 67]. In contrast to polyubiquitination-induced, proteosome-mediated degradation, monoubiquitylation leads to different functional outcomes of Smad proteins. The monoubiquitylation of r-Smad2 by Itch (Itchy E3 ubiquitin protein ligase) promotes the stable interaction between r-Smad2 and TGFβRI. However, the monoubiquitylation of r-Smad3 interferes with Smad3’s ability to bind to target loci [68, 69].

TGF-β signaling pathways also regulate gene transcription epigenetically by interacting with chromatin remodelers, histone modifiers, DNA modifiers, chromatin readers, and long noncoding RNAs [70]. r-Smad2 interacts with SMARCA4 (SWI/SNF related, matrix associated, actin dependent regulator of chromatin, subfamily A, member 4; also known as BRG1) to epigenetically modify chromatin structures for the expression of a majority of the TGF-β-targeted genes, except i-Smad7 and SnoN, to achieve a tightly controlled feedback loop of TGF-β signaling [71]. HAT (histone acetyltransferase) and HDAC (histone deacetylase) are importantly involved in TGF-β signaling [72]. r-Smad1, r-Smad2, r-Smad3, r-Smad5, and co-Smad4 can all recruit HAT or HDAC through the MH1 domain to control target gene expression in a context-dependent manner [73–75]. In addition to modifying histone acetylation, Smad proteins regulate histone methylation. In response to Nodal stimulation, r-Smad2 and r-Smad3 recruit histone demethylase JMJD3 (Jumonji domain-containing protein 3) to the promoter region to reduce inhibitory histone markers of H3K27me3 [76].

### TGF-β signaling through Smad-independent pathways

In addition to Smad-dependent pathways, TGF-β receptors can signal through Smad-independent pathways, including ERK (extracellular signal-regulated kinase), JNK, p38 MAPK, PI3K (phosphatidylinositol-3 kinase), AKT, and Rho family GTPases [48, 77] (Fig. 1). TGF-β-activated ERK signaling requires ShcA. Activated TGFβRI recruits and phosphorylates ShcA on tyrosine and serine residues. Phosphorylated ShcA assembles the ShcA-Grb2-SOS complex and activates Ras for MEK1/2 and ERK signaling [78]. Activated TGFβR recruits TAK1 (TGF-β activated kinase 1) to further activate the p38 MAPK and JNK pathways. TGF-β stimulates the PI3K-AKT-mTOR pathway through an indirect association of activated TGFβRI and TGFβRII with p85, a regulatory unit of PI3K [79]. TGF-β can also induce the ubiquitination of p85 through TRAF6, resulting in AKT-mTOR activation [80], which is important for cell proliferation, metabolism, and migration [81, 82].

TGF-β signaling has evolved to carry out broad functions through diverse mechanisms by integrating various environmental stimuli in a cell-type- and context-dependent manner. Since TGF-β and its signal are central to a myriad of cellular functions that are vital for development, health, and disease, TGF-β and its signal must be carefully regulated. Such regulation is achieved by imposing controls at every step of the signaling process to ensure proper outcomes in response to the unique combination of stimuli in a specific niche. In the following sections, we will discuss the roles of TGF-β signaling in regulating Treg and Th17 cells. We will first introduce the important aspects of the biology of Treg and Th17 cells and then discuss in detail how TGF-β signaling regulates their functions.

## T_REG_ Cells are central to immune tolerance and homeostasis

Approximately a century ago, the concept that mechanisms must exist to prevent autoimmunity and to uphold tolerance was proposed by Paul Ehrlich, albeit in an absolute sense denying the possibility of autoimmunity. The very existence of autoimmunity was not accepted until after the 1960s [83]. With the realization that self-tolerance is not given and can be broken to result in autoimmune diseases, much effort has been devoted to understanding how tolerance is established and maintained. Decades of investigation have informed us that multiple mechanisms contribute to tolerance, including clonal selection, anergy induction, and active immune suppression through cells and cytokines [84–87]. In the 1970s, the existence of a cell type with active immune suppression function was proposed [88–90]. It was not until the 1980s that Sakaguchi et al. identified Lyt-1^high^,2,3^low^ T cells enriched for immune-suppressive activity [91, 92]. In 1995, a seminal work revealed that CD4^+^CD25^+^ cells are markers for a T-cell population that is highly enriched for immune-suppressive function [93], allowing for extensive characterization of these cells.

The genetic underpinning of suppressor T cells was revealed in the early 2000s. Monogenetic loss-of-function mutation of an X-linked gene called *Foxp3* (forkhead box p3) was found to be accountable for multifocal lymphoproliferation autoimmunity syndrome in scurfy mice and IPEX (immunodysregulation, polyendocrinopathy, and enteropathy, X-linked) human patients [94–97]. It was later found that the *Foxp3* gene is predominantly, if not exclusively, expressed in CD4^+^CD25^+^ T cells [98] and that Foxp3 is sufficient and required for the generation and function of CD4^+^CD25^+^ suppressor T cells [99, 100]. CD4^+^CD25^+^ T cells with Foxp3 expression and immunosuppressive activity are now defined as regulatory T (Treg) cells. Of note, while Foxp3 expression alone can mark mouse Treg cells with good certainty, it is often unreliable for human Treg cells, as Foxp3 can be upregulated, often transiently, in activated human CD4^+^ T cells with no suppressive function [101–103]. In this review, we will focus on discussing mouse Treg cells.

### Treg cell classification, generation, and maintenance

Treg cells can develop in the thymus. Thymus-derived Treg cells are known as tTreg cells. In addition, Treg cells can be generated extrathymically at peripheral sites. Periphery-generated Treg cells are known as pTreg cells. Moreover, Treg cells can be induced in cell culture when CD4^+^ T cells are activated in the presence of TGF-β. TGF-β-induced Treg cells in culture are known as iTreg cells [104, 105]. The expression of *Foxp3* is induced in tTreg cells during thymic development in response to moderately strong TCR stimulation in conjunction with interleukin-2 (IL-2) produced by activated self-reactive thymocytes [106–109]. tTreg cells stably express *Foxp3* and other Treg cell markers by extensive epigenetic modification at relevant genetic loci for systemic self-tolerance [110]. pTreg cells can be generated from Foxp3^–^ CD4^+^ T cells that are exposed to factors, including TGF-β and IL-2, in peripheral tissues [111, 112]. These pTreg cells accumulate mostly at barrier sites (such as the intestines) where they respond to innocuous antigens derived from food or commensal microbes, metabolites, and environmental factors [113–129]. Non-Treg cells can be induced to become iTreg cells in culture by TGF-β [130], retinoic acid [122], and IL-2 [131] following TCR stimulation. While iTreg cells can express high levels of Foxp3 and are immunosuppressive, they generally lack the epigenetic modifications of tTreg cells to stabilize Treg cell phenotypes [132, 133]. In addition to the populations of Foxp3^+^ Treg cells defined by the above taxonomy [105], Treg cells can be further or alternatively categorized into naive, activated, effector, and memory Treg cells based on the surface expression of CD62L, CD44, CD127, CD69 and Klrg [134]. Tissue-resident Treg cells have distinct genetic programs from tTreg cells and bear unique markers, including CD103 and chemokine receptors, to regulate immune responses in a tissue-specific manner [134, 135].

**Fig. 2:**
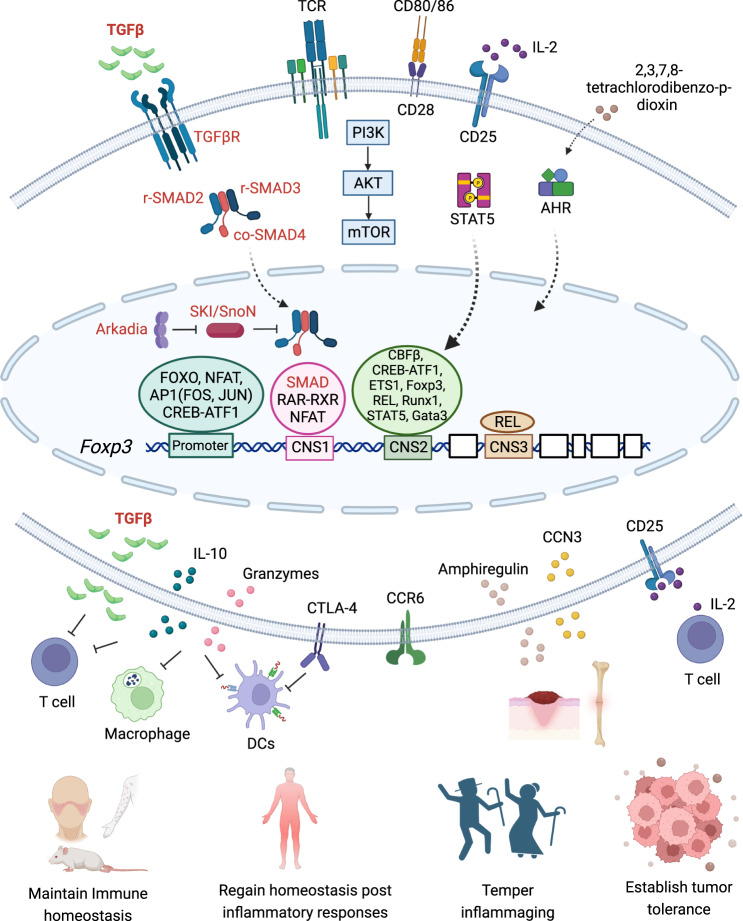
TGF-β signaling promotes Treg cell function for tolerance and homeostasis. TGFβR and Smad proteins promote Foxp3 expression and Treg cell generation by activating CNS1 of the *Foxp3* locus. The promoter, CNS2, and CNS3 regions are also important for the induction and stability of *Foxp3* expression by integrating a myriad of upstream signaling pathways, including TCR, CD28, CD25, AHR and various transcription factors. The Arkadia/SKI/SnoN pathway is important for TGF-β-induced *Foxp3* expression. Foxp3-expressing Treg cells suppress the function of T cells, macrophages, and DCs through TGF-β, IL-10, granzymes, CTLA-4, and CD25. Treg cells promote tissue repair through CCR6-mediated chemotaxis and amphiregulin- and CCN3-mediated tissue regeneration to maintain tolerance and homeostasis in health and disease

Stable Foxp3 expression is important for Treg cell maintenance, identity, and function [136–139] (Fig. 2). Cytokines are central to Treg cell homeostasis. γ_c_-dependent cytokines are critical for the development and homeostasis of tTregs as well as pTreg and iTreg cells. TGF-β is especially important for inducing pTreg and iTreg cells [109, 140]. In addition, PI3K-mTOR-AKT, an important signaling axis regulated by TCR, costimulation, and cytokines, including TGF-β, plays a pivotal role in the development, function, and maintenance of Treg cells by regulating immune metabolism [141, 142] and the expression of genes critical for Foxp3 expression, including FOXOs [143–145]. Mechanisms also exist to specifically control stable Foxp3 expression independent of its induction. Three CNSs (conserved noncoding DNA sequences), namely, CNS1-3, in *Foxp3* loci are critical for *Foxp3* expression [146]. While CNS1 and CNS3 are important for TGF-β- and TCR/CD28-induced *Foxp3* expression, respectively, CNS2 is specifically required to stabilize *Foxp3* expression in the progeny of dividing Treg cells. Foxp3, Runx1-Cbfβ, and GATA3 bind to CNS2 to promote stable *Foxp3* expression [146–149]. In addition, the posttranslational modifications of Foxp3 proteins, including phosphorylation, O-GlcNAcylation, acetylation, ubiquitylation, and methylation, are important for controlling the functions of Foxp3 and therefore Treg cells [150, 151]. It is thus predicted that perturbations in the abovementioned mechanisms through genetic, pharmacological, and environmental means alter Treg cell homeostasis and function, with or without affecting the stability of Foxp3 expression [152–160].

### The mechanisms of Treg cell function

Treg cells control adaptive and innate immune responses through broad mechanisms in both antigen-specific and antigen-nonspecific manners [161] (Fig. 2). Treg cells carry out their functions through cytokine modulation, cytolysis, metabolic disruption, and modulation of DC maturation and function [140, 162]. Treg cells can directly suppress T-cell function by acting as a sink for IL-2, which is critical for the survival and proliferation of activated effector T cells [163, 164], as CD25 (IL2Ra) is highly expressed by Treg cells. Treg cells, especially activated Treg cells, produce large amounts of TGF-β and IL-10 to suppress both adaptive and innate immunity [84]. High levels of CTLA-4 (cytotoxic T-lymphocyte associated protein-4) on the surface of Treg cells can downregulate costimulatory molecules on antigen-presenting cells (APCs) and induce tolerogenic DCs [165, 166] to reduce the ability of APCs to activate non-Treg cells [101, 167]. Treg cells produce elevated levels of granzymes that are suggested to target APCs for disruption [168]. Treg cells are also found to form more stable interactions with APCs than non-Treg cells [169, 170] to prevent non-Treg cells from interacting with APCs. Of note, Foxp3 reprograms Treg cell metabolism to adapt to low-glucose, high-lactate environments [171]. This allows Treg cells to utilize different energy resources than other activated immune cells for better fitness in a specific niche, especially under inflammatory conditions [172, 173]. Treg cells migrate to and reside in a specific niche through chemotaxis by sensing chemokine gradients and expressing relevant chemokine receptors and integrins [174–176]. Interestingly, the molecular program of Treg cells seems to be quite flexible and heterogeneous to allow Treg cells to tailor their function to suppress specific responses. Treg cells can express T-bet, a Th1 cell differentiation factor [177], with an enhanced ability to suppress Th1 responses [178]. Treg cells can express IRF4 (interferon regulatory factor 4), a Th2 differentiation factor [179], with an enhanced ability to suppress Th2 responses [180]. Treg cells can express RORγt, a Th17 differentiation factor [181], with an enhanced ability to suppress Th17 responses [182]. The mechanisms for these observations are not entirely clear. Two mutually nonexclusive possibilities are that (1) existing tTreg cells and/or residential Treg cells activated under Th1-, Th2-, and Th17-skewing conditions adopt respective molecular programs upon activation, as the epigenetic program of Th differentiation of Treg cells is “poised and flexible” but not “fixed” to allow such adaptation [157–160, 183, 184]. Adapted Treg cells, likely antigen specific, become better fitted in a microenvironment with enhanced suppressive function toward ongoing responses. (2) In a specific microenvironment with ongoing inflammation, a fraction of antigen-specific non-Treg cells are converted into pTreg cells by acquiring Foxp3 expression and suppressive function while maintaining their Th program acquired before conversion. These converted pTreg cells will likely have enhanced function toward ongoing responses. In fact, studies have shown that a fraction of CD4^+^ T cells, or pTreg cell precursors, in the intestines express RORγt before acquiring Foxp3 and Treg cell properties [185, 186].

While the cardinal function of Treg cells is to suppress immune responses, Treg cells are also important for promoting tissue repair through both indirect and direct mechanisms. The indirect mechanism can be attributed to the suppressive function of Treg cells [187]. Inflammatory cells, including neutrophils, macrophages, and activated conventional T cells, infiltrate into the site of inflammation and release tissue-damaging cytokines to adversely affect tissue repair. By suppressing the effector function of inflammatory cells, Treg cells promote tissue repair indirectly [187, 188]. More importantly, Treg cells can promote tissue repair through direct, immune-suppression-independent mechanisms. During inflammation, activated Treg cells migrate to inflammatory tissues, a process that requires CCR6, a chemokine receptor [189–191]. Tissue-infiltrating Treg cells produce amphiregulin, the epidermal growth factor receptor (EGFR) ligand, to promote tissue repair in acutely injured skeletal muscle and in acutely influenza virus-infected lungs [192–194]. In addition, CCN3, a growth regulatory protein implicated in tissue regeneration, is produced by Treg cells to promote oligodendrocyte differentiation and myelination to facilitate CNS tissue regeneration and to ameliorate neuro-immune pathologies, including EAE and MS [195, 196]. The myriad of Treg cell functions in immune suppression and tissue repair enable the broad involvement of Treg cells in health and disease.

#### Maintaining immune homeostasis

Immune homeostasis is the state in which the immune system maintains a balance between immune activation and immune tolerance. As one of the pillars of tolerance, proper Treg cell function is central to immune homeostasis [161, 197, 198]. Dysregulation of Treg cell function caused by genetic or environmental factors invariantly disrupts immune homeostasis and tolerance and often causes autoimmunity [199]. Therefore, enhancing Treg cell function can benefit disease treatment. For instance, low-dose IL-2 has been used to treat T1D by specifically promoting Treg cell function [200, 201]. More recently, adoptive Treg cell therapy has gained much attention for the treatment of autoimmune diseases in an antigen-specific manner [202–204]. In graft-versus-host or host-versus-graft diseases that can be viewed as a form of autoimmunity introduced through transplantation, enhancing Treg cell function benefits transplant tolerance [205, 206]. During allergic diseases resulting from tolerance breakdown mostly due to environmental factors, promoting Treg cell function helps to broadly restrain hyperactive T cells, eosinophils, mast cells, basophils, antibody isotype switching, inflammatory DCs, and inflammatory cell migration to tissues [207].

In addition to preventing and restraining autoimmune inflammation, Treg cells are important for establishing and restoring homeostasis during host–microorganism interactions. During the host‒pathogen interaction and pathogen clearance response, the activation of innate and adaptive immunity by pathogens causes inflammatory and immune pathology in hosts. Excessive immune activation often causes pathologies that can be debilitating or lethal. Proper Treg cell function is important to restore immune homeostasis during and after pathogen clearance. Treg cell-mediated suppression is necessary to temper inflammatory responses during infection and regain immune homeostasis after infection and pathogen clearance [208–210]. In addition, Treg cells establish tolerance to obligate microbiota [211]. Treg cells can achieve this by actively suppressing both innate and adaptive immunity through antigen-dependent and antigen-independent mechanisms [162] and by promoting tissue repair [187].

In addition to autoimmune and infection-induced perturbation of immune homeostasis, the aging process is often associated with a systemic inflammatory syndrome, also known as inflammaging. Inflammaging is a naturally occurring inflammation that progresses with age and contributes to immune senescence, immunological aging, and age-associated morbidities and mortalities, including infection, cancer, and autoimmunity in the elderly [212–214]. While many factors can contribute to inflammaging, reduced Treg cell function has recently been associated with inflammaging [215]. Aged Treg cells are more severely senesced and less proliferative than aged non-Treg cells. As a result, aged Treg cells are not optimal in restraining non-Treg cell function. Treg and non-Treg cell functions are off-balanced during aging, which may contribute to inflammaging [215]. Therefore, bolstering Treg cell function will help to establish, maintain, and restore immune and tissue homeostasis under normal and pathological conditions.

#### Establishing tumor tolerance

Tumors develop when the immune system fails to eradicate tumor cells. One reason for such a failure is due to the immune-suppressive TME (tumor microenvironment) established during coevolution of the tumor cells and the host. Treg cells are enriched in many tumor types and contribute to the TME and tumor tolerance [216–221]. Treg cells can be induced by self- and tumor-antigens in the presence of TGF-β, which is often produced by transformed cells, and can be clonally expanded in the TME [222, 223], aided by the Treg cells’ unique ability to metabolically adapt to low-glucose and high-lactate environments in tumors [171]. In addition to inducing Treg cells, tumors attract Treg cells by secreting chemokines, including CCL1, CCL5, CCL22, and CCL28, as well as by inducing chemokine receptor expression on Treg cells [220]. Strategies to target Treg cell generation, recruitment, and function promise to benefit cancer immunotherapy [224–226].

Treg cells impose critical and broad functions for tolerance, immune homeostasis, and tissue repair in health and disease. Much research has been focused on understanding how their generation and function are controlled. TGF-β emerges as a central regulator of Treg cell biology. In the following section, we will discuss in detail how TGF-β signaling controls various aspects of Treg cell biology in health and disease.

## TGF-β controls T_REG_ Cell generation, homeostasis, and function through cell-type and context-dependent mechanisms

### The varying roles of TGF-β in Treg cell generation and homeostasis under different contexts

The interest in TGF-β in Treg cell function stems from early studies demonstrating that TGF-β1 deletion led to a multifocal lethal inflammatory disease early in life in mice in the 1990s [21–23]. The ensuing studies revealed that TGF-β is an immune regulatory cytokine that inhibits activation-induced T-cell proliferation and more potently suppresses Th cell differentiation and effector function [227]. The relationship between TGF-β and Treg cells became clear when a seminal study revealed that TGF-β promotes CD4^+^CD25^-^ conventional T cells to differentiate into CD4^+^CD25^+^ cells upon activation in culture [130]. TGF-β-induced CD4^+^CD25^+^ cells are anergic and do not produce Th1 or Th2 cytokines, yet these cells express Foxp3 and produce TGF-β. Importantly, TGF-β-induced CD4^+^CD25^+^ cells are immunosuppressive and inhibit antigen-driven CD4^+^ T-cell expansion during lung inflammation [130]. Therefore, under culture conditions, TGF-β is sufficient to induce the generation of Treg cells that phenotypically resemble Treg cells identified in vivo. A later study using a Foxp3-mRFP reporter mouse strain found that such an induction occurs by promoting de novo Foxp3 expression in activated naive CD4^+^ T cells [132]. One mechanism by which TGF-β promotes Foxp3 expression is through r-Smad3 and NFAT (nuclear factor of activated T cells) binding to a Foxp3 enhancer region in T cells [228]. This enhancer region was later found to be the CNS1 region [146]. Other mechanisms can be through downregulating inhibitory i-Smad7 at the transcriptional level through Foxp3, which results in boosted TGF-β signaling through enhancing the r-Smad3 and co-Smad4 response to promote Foxp3 expression [229], suggesting that TGF-β promotes iTreg cell generation through a self-enhancing feedforward mechanism. While naive T cells are readily converted into Treg cells by TGF-β stimulation after activation in culture, TGF-β is incapable of converting predifferentiated Th cells into iTregs in culture [230], which could be due to the altered molecular context in differentiated Th cells and reduced TGFβRI expression in activated T cells [231].

Because TGF-β signaling can promote Foxp3 expression and Treg cell function both in culture and in vivo, whether and how TGF-β pathways are required for Treg cell function in vivo are important questions to be addressed. Studies were conducted in which components of TGF-β signaling pathways in T cells were deleted under various conditions. In one study, T-cell-specific knockout of TGFβRII led to reduced Treg cells in the periphery but not in the thymus [35]. In a mixed bone marrow chimeric mouse model, where both wild-type and TGFβRII-knockout T cells coexist, TGFβRII was later found to be required for maintaining Treg cells in the periphery in a cell-intrinsic manner [232]. Detailed analysis of T-cell-specific TGFβRI knockout mice revealed that TGFβR is critical for tTreg cell generation in the thymus [33]. Such a TGF-β-mediated effect can occur through a direct mechanism because TGF-β is abundant in the thymus due to thymocyte apoptosis during thymic selection [233]. The defective tTreg cell generation due to TGFβRI deletion can be compensated for, however, by the enhanced IL-2 response of TGFβRI-deficient Treg cells under inflammatory conditions in these mice [33]. Another mechanism by which TGFβR is required for tTreg cell generation may be indirect, through increased death of TGFβR-deficient tTreg cells under inflammatory conditions [234]. IFN-γ, an inflammatory cytokine suppressed by TGF-β, impairs the homeostasis of TGFβR-deficient Treg cells. IFN-γ deletion largely restored the TGFβRII-deficient Treg cell population in the periphery in a BDC2.5 NOD mouse model [235]. Therefore, under inflammatory conditions, TGF-β promotes Treg cell generation and maintenance in the thymus and periphery through both direct and indirect mechanisms.

The aforementioned findings also provide further understanding of how TGF-β may be involved in Treg cell generation and homeostasis in the absence of inflammation. Available evidence suggests that TGFβR is dispensable for the maintenance and function of existing Treg cells under homeostatic conditions. Unlike deleting floxed *Tgfbr2* alleles in developing thymocytes using a *Cd4-Cre* transgene, deleting floxed *Tgfbr2* alleles in mature T cells in the periphery using a *distal-Lck-Cre* transgene does not lead to autoimmunity or lymphoproliferation under steady state [236]. These findings indicate that lymphopenia-driven T-cell proliferation and inflammation are restrained by TGF-β signaling, which is important for Treg cell survival. In agreement, Treg cell-specific deletion of TGFβRII did not lead to systemic inflammation and did not apparently perturb tTreg cell populations [233, 237]. It is therefore plausible that TGF-β is critical for tTreg cell generation and maintenance in a context-dependent manner, depending on the inflammatory status of the niche. Further studies are warranted to understand why TGFβR is important for the generation and maintenance of tTreg cells, especially under inflammatory conditions.

The finding that TGFβR is required for the generation and maintenance of Treg cells in a context-dependent manner prompts the question of what sources of TGF-β are critical for Treg cell homeostasis in vivo. TGF-β is broadly produced by many cell types, including immune cells and nonimmune cells, in a localized manner. Of interest, T cells, especially Treg cells, produce TGF-β1 in a membrane-bound form [238, 239]. Deletion of TGF-β1 specifically in T cells or in Treg cells does not lead to early-onset autoimmunity, unlike in T-cell-specific TGFβR knockout mice [239, 240], although T-cell-specific TGF-β1 knockout mice develop immunopathology later in life [240]. Nonetheless, Treg cell homeostasis is not obviously perturbed in these mice. In fact, the tTreg population is slightly increased, suggesting that endogenously generated TGF-β1 by tTreg cells restrains tTreg cell homeostatic proliferation but is dispensable for their generation or maintenance under noninflammatory, homeostatic conditions. A study, however, challenges these findings by showing that the deletion of TGF-β1 in Treg cells led to impaired Treg cell homeostasis and autoimmune syndrome in mice [241]. Further examination revealed that the findings in this study were due to cryptic genetic manipulation, which led to undue Treg cell death [242]. Thus, the notion that TGF-β1 is dispensable for Treg cell homeostasis stands. Nonetheless, in TGF-β1^−/−^ mice, where systemic inflammation occurs, TGF-β1 was shown to be important for Treg cells to be maintained in the periphery and to maintain the stability of Foxp3 expression [243]. Therefore, it becomes obvious that TGF-β controls Treg cell generation and homeostasis in a context- and microenvironment-dependent manner; while TGF-β is largely dispensable for Treg cell homeostasis under noninflammatory conditions, inflammation makes Treg cells sensitive to the loss of TGF-β and its signal for their generation, maintenance, and stability. The mechanisms underlying such a dichotomous role of TGF-β in Treg cells need further investigation to understand how Treg cells can be maintained under inflammatory conditions when needed most.

### Multilayered mechanisms of TGF-β-promoted Treg cell generation and homeostasis

While TGF-β promotes both tTreg and iTreg cell generation in a context-dependent manner, TGF-β has differential effects on the genetic programs of tTreg and iTreg cells. Some of the tTreg genes, including *Il2ra*, *Socs2*, *Tnfrsf18*, and *Ctla4*, are not enhanced by TGF-β [244]. iTreg cells rely on continuous TGF-β signaling to maintain Foxp3 expression, without which they have a brief life and reduced Foxp3 stability when compared with Treg cells isolated in vivo [245]. While r-Smad2 and r-Smad3 are required for the generation of iTreg cells induced by TGF-β [246, 247], tTreg cell generation and homeostasis are unperturbed when r-Smad2 and r-Smad3 are deleted [246–249]. In addition to r-Smad2 and r-Smad3, the Smad-independent, TAK1-dependent TGFβR signaling pathway contributes to tTreg cell homeostasis [248]. Similarly, T-cell-specific knockout of co-Smad4 does not obviously affect tTreg cell generation even when TGFβRII is absent. However, iTreg cell generation is severely reduced when co-Smad4 is deleted [232, 250]. T-cell-specific knockout of Arkadia, an E3 ligase that mediates the degradation of i-Smad7, SKI, and SnoN, which are inhibitory to TGF-β signaling, led to reduced generation of iTreg cells in culture and decreased generation of intestinal RORγt^+^Foxp3^+^ pTreg cells but not the generation of tTreg cells [251]. One of the mechanisms underlying these findings could be through co-Smad4, as co-Smad4 is required for iTreg cell generation [232, 250], and the function of co-Smad4 is suppressed by i-Smad7 [252], SKI, and SnoN [67, 253]. The notion that TGF-β functions differently in tTreg, pTreg and iTreg cells is further supported by the findings that CNS1 is critical for iTreg and pTreg cell generation but can be compensated for by CNS3 in tTreg cells [146]. It was also reported that mice lacking CNS1 generated tTreg cells normally but generated fewer pTreg cells in the intestines [254]. These findings suggest that the generation of tTreg cells and pTreg and iTreg cells utilize distinct mechanisms for Foxp3 expression: TGF-β signaling is important for pTreg and iTreg cells but is much less important for tTreg cell generation under noninflammatory, homeostatic conditions. While TGF-β1 is the predominant cytokine that can promote iTreg cell generation, Activin A, another TGF-β superfamily member, can also promote iTreg cell differentiation in culture by synergizing with TGF-β1 but not on its own, indicating an interaction between TGF-β and Activin A in controlling Treg cell function. Activin A does so by promoting Smad and p38 MAPK signaling initiated by TGF-β [255, 256]. How important Activin A is in the biology of tTreg and pTreg cells in vivo remains to be addressed. In addition, whether other members of the TGF-β superfamily are also involved in Treg cell generation needs to be elucidated since their signaling can impact co-Smad4 function, which is critically involved in iTreg cell generation.

### Involvement of TGF-β in Treg cell function

The role for TGF-β in regulating Treg cell function has been under debate. The first connection between TGF-β and Treg cell suppressive function was from a study using a T-cell transfer-induced colitis mouse model. In this study, the suppressive function of Treg cells was found to be abrogated by anti-TGF-β1 antibodies [257]. In addition, it was found that Treg cells mediate their suppressive function through surface-bound TGF-β1 in culture [258]. Since then, various TGF-β- and TGFβR-deficient (TGF-β1^−/−^, TGFβRI^−/−^, and TGFβRII^−/−^) mouse models have been generated to investigate the relationship between Treg cell function and TGF-β. TGF-β1 was found to be indispensable for Treg cell-mediated function [240]. TGF-β1^−/−^ Τreg cells failed to suppress the colitis induced by cotransferred naive wild-type CD4^+^ T cells, which displayed enhanced Th1 cell differentiation in recipient mice [240]. However, it was later found that Treg cell-specific knockout of TGF-β1 did not lead to systemic inflammation and had a negligible effect on immune homeostasis and EAE development [239]. These findings suggest that while Treg cell-produced TGF-β is dispensable for immune homeostasis under steady-state conditions, Treg cell-produced TGF-β may become important under certain inflammatory conditions. Of interest, a study where TGFβRI is specifically deleted in Treg cells revealed that TGF-β signaling is important for certain functions of Treg cells [237]: TGFβRI deletion in Treg cells leads to reduced T-bet but increased RORγt expression and showed an increased ability to suppress Th1 cells but reduced control of Th17 cells in aged mouse lungs and GI tracts and during EAE in young mice. In addition, TGFβRI is critical for Treg cell recruitment and retention in the gastrointestinal tract. TGFβRI deletion in Treg cells leads to reduced expression of the tissue residential protein CD103 and colon chemotaxis protein GPR15 and increased expression of GPR174, a LysoPS (lysophosphatidylserine) receptor that negatively regulates Treg cell homeostasis [259], limiting Treg cell accumulation in the intestines [237]. This study reveals a tissue-specific role of TGF-β signaling in controlling Treg cell function, emphasizing how TGF-β may control Treg cells in a microenvironment-dependent manner. While TGF-β promotes the expression of amphiregulin in fibroblasts [260], whether and how TGF-β signaling is indeed involved in Treg cell-mediated tissue repair remains to be addressed.

Collectively, available evidence suggests that TGF-β and related signals are not always essential for Treg cell generation, homeostasis, or function under all circumstances, as initially thought. Nonetheless, TGF-β and related signals are indeed required for iTreg cell differentiation in culture and pTreg cell generation in vivo, and such a requirement becomes inflammation-dependent for tTreg cells. Therefore, TGF-β and related signals are critical for Treg cells in a cell-type- and context-dependent manner (Fig. 2). Much study is needed to fully understand how TGF-β signaling controls the generation and function of various Treg cell subsets through genetic and epigenetic mechanisms at the genomic and proteomic levels with or without crosstalk with other factors that sense complex environmental cues.

## Th17 cells are broadly involved in immune pathogenicity and regulation

In the 1980s, Mosmann and Coffman’s work established a paradigm that, in response to different cytokines, naive CD4^+^ T cells may differentiate de novo into functionally distinct effector Th cells, namely, IFN-γ-producing Th1 cells and IL-4-producing Th2 cells, to regulate different immune responses [261]. A later study found that the p40 subunit of IL-12, a Th1 cell differentiating factor, not only pairs with the p35 subunit to form IL-12 but also pairs with the p19 subunit to form IL-23 [262]. Therefore, observations made by targeting p40 may not be entirely attributed to the function of IL-12 and thus Th1 cells. Indeed, it was later found that IL-23 but not IL-12 is crucial for the induction of autoimmune EAE (experimental autoimmune encephalomyelitis) [263]. Subsequent studies revealed that IL-23, but not IL-12, promoted an IL-17-producing T-cell population, now called Th17 cells, in a p19-dependent manner [264–266]. Th17 cells are distinct from Th1 cells.

### Th17 cell classification, generation, and maintenance

**Fig. 3:**
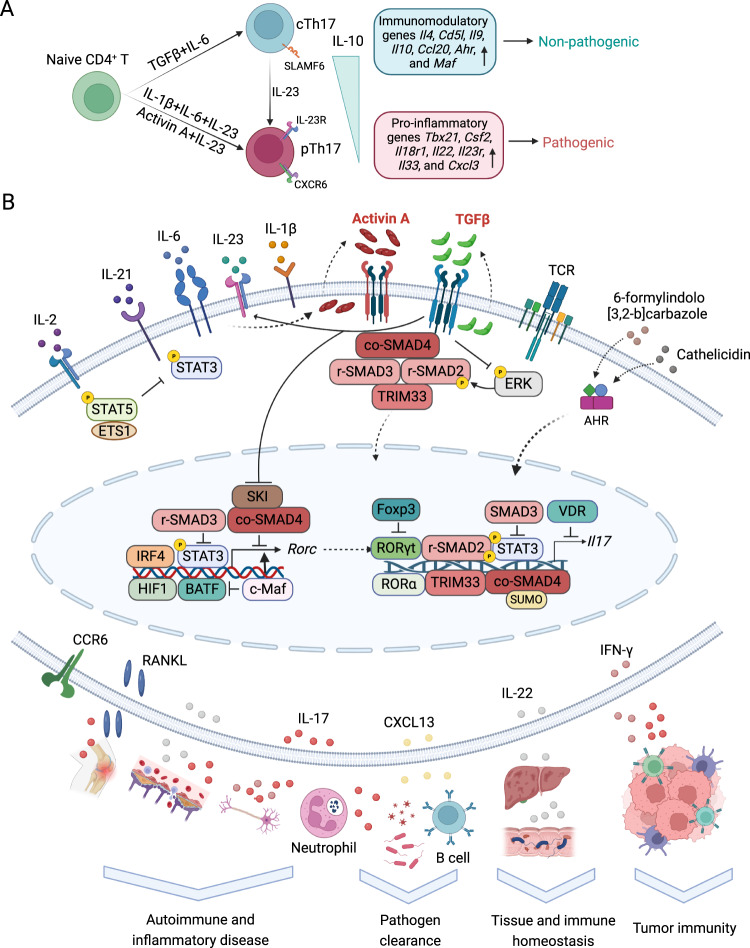
TGF-β superfamily signaling controls the biology of Th17 cells with broad functions in health and disease. **A** Upon culturing in the presence of different cytokine combinations, activated CD4^+^ T cells can be differentiated into Th17 (pTh17) cells of high pathogenicity and Th17 (cTh17) cells of low pathogenicity. cTh17 and pTh17 cells bear different molecular signatures. **B** By integrating various signaling pathways, including TCR, interleukins, and AHR, the TGF-β superfamily members TGF-β and Activin A control RORγt expression and function as well as *Il17* expression through Smad and interacting proteins during Th17 cell differentiation. Differentiated Th17 cells regulate immunity, autoimmunity, cancer, and homeostasis through the secretion of cytokines

Th17 cells have diverse, sometimes opposite, functions. Th17 cells promote inflammation and autoimmune diseases, clear pathogens, and maintain barrier function of the mucosa [267, 268]. Therefore, Th17 cells can be both pathogenic and nonpathogenic. The dichotomous function of Th17 cells appears to be dictated by the microenvironment in which they reside. Nonpathogenic Th17 cells accumulate in the intestines under homeostasis to maintain mucosal integrity. Pathogenic Th17 cells can accumulate in the skin, nervous system, and skeleton during immune pathologies, including psoriasis, MS (multiple sclerosis), RA (rheumatoid arthritis), and AS (ankylosing spondylitis) [269]. In addition, intestinal nonpathogenic Th17 cells have been shown to be stem cell-like precursors for pathogenic Th17 cells in the spinal cord that cause EAE [270]. Therefore, the generation and maintenance of stem cell-like, nonpathogenic Th17 cells in vivo appears to depend on the microenvironment in the intestines, without which Th17 cells differentiate into pathogenic Th17 cells by default. The functional dichotomy of Th17 cells can also be observed in Th17 cells differentiated in culture. The TGF-β1 + IL-6 cytokine combination induces IL-10-producing Th17 cells with less pathogenic function [271]. IL-1β + IL-6 + IL-23, TGF-β + IL-6 + IL-23, and Activin-A + IL-6 cytokine combinations induce Th17 cells of potent pathogenic function with much less IL-10 production [269, 272, 273]. In agreement, nonpathogenic Th17 cells and pathogenic Th17 cells have different gene expression patterns. Pathogenic Th17 cells express proinflammatory genes, including *Tbx21*, *Csf2*, *Il18r1*, *Il22*, *Il23r*, *Il33*, and *Cxcl3*. In contrast, nonpathogenic Th17 cells express high levels of immunomodulatory genes, including *Il4*, *Cd5l*, *Il9*, *Il10*, *Ccl20*, *Ahr*, and *Maf* [121, 269, 274, 275] (Fig. 3 A). Interestingly, IL-23 can convert nonpathogenic Th17 cells into pathogenic Th17 cells [274]. Therefore, the first-identified Th17-driven cytokine, IL-23, is not only important in promoting Th17 cell proliferation and survival [276] but also in endowing Th17 cells with pathogenic function. Single-cell RNA-seq analysis of Th17 cells isolated from EAE-diseased mice revealed that *Il10* and *Cd5l* are nonpathogenic Th17 cell markers that are coexpressed with proinflammatory genes, including *Gpr65*, *Toso*, and *Plzp* [277, 278]. It therefore appears that nonpathogenic Th17 cells have “dual intentions”, agreeing with the observation that these cells have the properties of stem cells and precursors [270]. In addition, pathogenic and nonpathogenic Th17 cells can be discriminated in EAE by GM-CSF^+^IFN-γ^+^CXCR6^+^IL-17^+^ and TCF1^+^SLAMF6^+^IL-17^+^, respectively [270].

Cytokines are critical to control, both positively and negatively, Th17 cell differentiation (Fig. 3B). IFN-γ and IL-4 are dispensable for Th17 cell differentiation. Instead, they inhibit the development of Th17 cells [265, 266]. The Th1 cell-polarizing cytokine IL-12 rapidly and irreversibly shuts down the *Il17a/f* locus in already-differentiated Th17 cells [275]. TGF-β can promote Foxp3 expression and thus Treg cell generation to suppress Th17 cell generation [250, 279, 280]. Nonetheless, TGF-β promotes Th17 cells in the absence of Foxp3 in vivo [186]. IL-6 and IL-21 promote Th17 cell differentiation through STAT3 [268, 281, 282]. IL-21’s function appears complex and context-dependent because when IL-21 signaling was blocked, the generation and function of Th17 cells were unaltered [283–285] during EAE but were reduced in gut inflammation [286]. Although IL-23 is not the differentiation factor for Th17 cells, productive and sustained Th17 cell responses only develop in the presence of IL-23, as revealed by studies with *Il23p19*^−/−^ and *Il23r*^−/−^ mice [263]. After the initial induction of Th17 cells, the availability of IL-23 becomes the limiting factor that determines whether Th17 cells, especially pathogenic Th17 cells, are sustained during the inflammatory response [287].

Combined signaling of TGF-β and IL-6/STAT3 and IL-21/STAT3 drives Th17 cell differentiation by promoting the expression of the transcription factors RORγt and RORα [181, 288–293], which in turn controls Th17 cell differentiation through shared and distinct mechanisms [294]. RORα and RORγt bind to the RORE (ROR response element) in the *Il17a/f* gene loci [291, 295] to directly promote IL-17 expression. STAT3 also binds directly to and transactivates the *Il17* and *Il21* promoters [296, 297]. Therefore, STAT3 and RORγt cooperate for Th17 cell generation. Some loci targeted by STAT3 in Th17 cells are also the targets of STAT5, a signal transducer of IL-2 that inhibits Th17 cell differentiation [298]. On these genetic loci, STAT3 promotes permissive histone modifications, yet STAT5 promotes repressive histone modifications.

In addition to RORs, other transcription factors are also important for controlling Th17 cell differentiation. IRF4, which is associated with the differentiation of the Th1 and Th2 cell subsets [299–301], is required for the differentiation of Th17 cells through RORγt-dependent and RORγt-independent mechanisms [302]. ETS1, the prototype member of the Ets family of transcription factors, inhibits Th17 cell differentiation by interacting with IL-2/STAT5 signaling because ETS1 deletion leads to increased Th17 cell differentiation with reduced IL-2 production and STAT5 signaling [303]. BATF (basic leucine zipper ATF-like transcription factor) and IRF4 form a complex to increase chromatin accessibility [304]. STAT3 then starts a transcriptional program that is eventually turned on by RORγt for Th17 cell differentiation [304]. Fosl2 limits the plasticity of Th17 cells [304]. c-Maf is importantly involved in Th17 cell generation in a context-dependent manner. c-Maf was found to function in a negative feedback loop to limit Th17 cell differentiation, where it is induced by both STAT3 and IRF4 and represses BATF [304, 305]. c-Maf is also critical for the maintenance, expansion, and function of differentiated Th17 cells by promoting the production of IL-21 [306] and RORγt [307].

Molecules other than cytokines also regulate Th17 cell differentiation. The host defense peptide cathelicidin promotes Th17 cell generation by enhancing AHR (aryl hydrocarbon receptor) and RORγt expression in a TGF-β1-dependent manner [308]. Although AHR activated by 2,3,7,8-tetrachlorodibenzo-p-dioxin induces Treg cells, AHR activated by 6-formylindolo[3,2-b]carbazole promotes Th17 cells to contribute to EAE [129, 244, 309]. Oxygen sensing appears important for Th17 cell differentiation because HIF-1 (hypoxia-inducible factor 1), a key metabolic sensor of oxygen, promotes Th17 cell differentiation through the direct transactivation of *Rorc* [310, 311]. Microbial and metabolic products, including retinoic acid, short-chain fatty acids, and bile acids, can also regulate Th17 cell differentiation [120–128, 147, 312]. Acetyl-CoA carboxylase, a key enzyme of de novo fatty acid synthesis, influences the Th17 and Treg cell balance through the glycolytic and lipogenic pathways [313]. CD5L, a signature marker of nonpathogenic Th17 cells, regulates lipidome saturation to restrain Th17 cell pathogenicity [278]. The active form of vitamin D (1,25-dihydroxyvitamin D3), the main ligand for the vitamin D receptor, has been found to “severely impair” the production of IL-17 and IL-17F by Th17 cells [314]. How cytokines and noncytokines deploy shared and distinct mechanisms to regulate Th17 cell differentiation is an important, albeit complex, question to be fully elucidated.

### All-encompassing Th17 cell function in health and disease

#### Contributions to autoimmune and inflammatory disease

Pathogenic Th17 cells contribute broadly to autoimmune and inflammatory diseases [268]. Th17 cells are important in causing the immune pathologies of diseases, including psoriasis [315], RA [316], MS [317], IBD (inflammatory bowel disease) [318], asthma [319, 320], Graves’ disease [321], transplant rejection [322, 323], and allergy [324]. T cells in human psoriatic skin lesions predominantly show a Th17 cell phenotype with high CCR6 expression [325]. This is in line with the observation that CCL20/CCR6 signaling is important for the chemoattraction of inflammatory cells to inflammatory tissues, including the skin. In RA patients, the expression of TNF, IL-1, and IL-17 is predictive of joint destruction [316]. Th17 cell-produced RANKL promotes RA by inducing osteoclastogenesis [326–328], which in turn promotes cartilage and bone destruction and resorption independently of TNF and IL-1 [329, 330]. In MS, IL-17 and IL-6 are among the most highly produced cytokines [317, 331]. In opticospinal forms of MS, IL-17 and CXCL8 (IL-8), a target of IL-17 and a strong neutrophil chemoattractant, are elevated and positively associated with spinal lesions [332]. Th17 cells effectively transmigrate across the blood‒brain barrier (BBB) and infiltrate into the CNS parenchyma through IL-17- and IL-22-mediated disruption of BBB tight junctions [333].

#### Promotion of pathogen clearance

Th17 cells play important roles in the pathogen clearance response, especially when type-1 and type-2 immunity are imbalanced [334]. Th17 cells traffic to the sites of infection due to high CCR6 expression. Diverse pathogens, including viruses, bacteria, and fungi-like microbes, can induce strong Th17 responses [335–352]. In humans, Th17 cells can recruit B cells through CXCL13 and promote antibody production by B cells for pathogen clearance [353–356].

#### Maintenance of tissue and immune homeostasis

In addition to being pathogenic for tissue damage, Th17 cells also promote tissue repair and homeostasis. Such an effect of Th17 cells can be mediated by IL-22, a member of the IL-10 family of cytokines [357], by promoting the regeneration of epithelial tissues [358]. During inflammation, Th17 cells migrate to the sites of inflammation via CCR6 [359, 360] and upregulate IL-22 [361] to limit liver damage in concanavalin A-induced hepatitis [362] and intestinal damage in T-cell-induced IBD [363]. Of note, IL-22 has also been found to be a pathogenic cytokine in psoriasis [364, 365] due to the IL-22-promoted excessive regeneration of epithelial cells. Thus, IL-22-producing Th17 cells may function in a cell-type- and context-dependent manner for tissue repair and homeostasis. Th17 cells, especially nonpathogenic Th17 cells, are important for establishing tolerance to commensal microbiota for homeostasis by coordinating with Treg cell functions [303]. In addition, the tissue repair function of Th17 cells will help maintain the barrier integrity of the mucosa to prevent microbes from translocating and the barriers from being breached [366–372]. Thus, Th17 cells in the mucosa are important for homeostasis by both preventing invasion of microbiota and promoting epithelial barrier integrity.

#### Participation in tumor immunity

Th17 cells and their cytokines are also involved in tumor development and cancer [373–377]. Th17 cells can differentiate into Th1-like Th17 cells (secreting both IFN-γ and IL-17) with stem cell-like properties to reject tumors [378, 379]. Compared with other Th cells, Th17 cells are long-lived with increased self-renewal ability. Such properties of Th17 cells seem to be due to their unique metabolic programming. Th17 cells utilize mitochondrial oxidative phosphorylation, which protects Th17 cells from apoptosis while enhancing their persistence in the periphery and TME [380]. Of note, Th17 cells have also been found to promote tumor formation induced by colonic inflammation in mice [381]. Therefore, the functions of Th17 cells in tumor development are complex and remain to be further characterized [382].

Th17 cells have both pathogenic and nonpathogenic properties. This allows Th17 cells to play broad and diverse roles in health and disease (Fig. 3B). The molecular mechanisms underlying the generation and function of Th17 cells have been under intensive investigation since their discovery. We now know that the TGF-β superfamily plays essential and discrete roles in controlling the biology of both pathogenic and nonpathogenic Th17 cells. In the following section, we will discuss in detail how TGF-β signaling pathways control Th17 cell function.

## TGF-β superfamily members deploy shared and unique mechanisms to control Th17 cell differentiation and function

### Importance of TGF-β in Th17 cell generation

During early studies defining Th17 cells as a new subset of Th cells that are proinflammatory and pathogenic and cause autoimmune neuroinflammation, TGF-β was not implicated in Th17 cell biology. Surprisingly, a seminal study demonstrated that TGF-β (a well-known immunosuppressive cytokine), in combination with IL-6, potently promoted IL-17 production and therefore Th17 cell differentiation under culture conditions [383–385]. This study demonstrated that TGF-β can promote inflammation by inducing IL-17 production [384]. The reconciliation of the seemingly contradictory roles of TGF-β in promoting both iTreg and Th17 cell differentiation in culture came from a later study showing that TGF-β balances Th17 and iTreg cell differentiation in a dose- and cytokine-milieu-dependent manner. In the lamina propria and during T-cell activation in the presence of TGF-β, Foxp3 and RORγt are coexpressed in CD4^+^ T cells and continuously counterbalance each other [279]. At low concentrations, TGF-β synergizes with IL-6 and IL-21 to favor Th17 differentiation by promoting IL-23R expression. However, high concentrations of TGF-β repress the expression of Th17 signatures, including IL-23R and IL-22, but promote the expression of Foxp3, which inhibits the activity of RORγt to favor iTreg differentiation [279]. In addition, Foxp3 antagonizes Th17 cell differentiation by inhibiting RORγt and RORα [250] and by interacting with Runx1 to suppress the *Il17* locus [280]. In agreement with the notion that TGF-β stimulation is compatible with Th17 cell differentiation, it was also found that TGF-β induces and maintains the expression of IL-6Rα, whose signaling suppresses Foxp3 expression and activates STAT3 to induce RORγt expression and Th17 cell differentiation [268, 384]. These findings suggest that while high concentrations of TGF-β promote Foxp3 expression to restrict the Th17 cell program, TGF-β nonetheless permits Th17 cell differentiation when IL-6 and IL21 are present. IL-6 and IL21 promote Th17 cell differentiation by enhancing the Th17 program and antagonizing the Treg cell program [386].

### Diverse functions of TGF-β signaling pathways in Th17 cell generation

Much effort has been devoted to understanding the molecular mechanisms through which TGF-β promotes Th17 cell differentiation. One study found that, in a dose-dependent manner, TGF-β suppresses the IL-6- and IL-21-promoted expression of SOCS3 (suppressor of cytokine signaling 3), a negative feedback inhibitor of STAT3. Interfering with TGFβR function with a dominant-negative form of TGFβII or the pharmacological TGFβRI inhibitor SB505124 leads to increased IL-6-induced SOCS3 expression and reduced STAT3 activation and thus fewer Th17 cells [387].

While TGF-β signaling is important for Th17 cell differentiation, the roles of the highly homologous r-Smad2 and r-Smad3 in Th17 cell differentiation seem distinct. r-Smad2 was found to positively regulate IL-17 expression by interacting with RORγt without affecting *Rorc* expression and to be required for Th17 cell differentiation [247]. T-cell-specific r-Smad2 knockout mice had reduced EAE with decreased Th17 cells [247]. In agreement, another study showed that Smad2 is important for Th17 cell generation, partly by regulating IL-6R expression [388]. Although closely related to r-Smad2, r-Smad3 was found to suppress Th17 cell differentiation by interfering with RORγt transcriptional activity [246]. r-Smad3 deletion led to increased RORγt expression and increased Th17 cell differentiation both in culture and in vivo, suggesting that r-Smad3 suppresses Th17 cell differentiation by interfering with the expression and function of RORγt [246]. Further study revealed additional mechanisms underlying the different roles of r-Smad2 and r-Smad3 in controlling Th17 cell differentiation. While r-Smad2 can be phosphorylated by ERK at the linker region and can associate with STAT3 and p300 as a coactivator to promote RORγt function, unphosphorylated r-Smad3 interacts with STAT3 and PIAS3 (protein inhibitor of activated STAT3) to repress *Rorc* and *IL17a* gene expression [389]. In addition, r-Smad2 is associated with TRIM33, a factor required for Th17 cell differentiation. TRIM33-deficient T cells developed less severe EAE [390]. TRIM33 associates with both *Il17a* and *Il10* loci in the presence of r-Smad2 to promote *Il17* expression and to suppress *Il10* expression, therefore facilitating pathogenic Th17 cell generation [390]. The Th17 cell-promoting function of r-Smad2 appears to dominate r-Smad3’s suppressive function because knocking out both r-Smad2 and r-Smad3 results in less Th17 cell differentiation without affecting RORγt expression [249]. These observations were, however, disputed by a study showing that neither r-Smad2 nor r-Smad3 alone is required for Th17 cell differentiation in culture and in EAE [391]. In addition, the combination of r-Smad2 deletion and r-Smad3 inhibition by a pharmacological inhibitor did not significantly affect Th17 cell differentiation [391]. Instead, Th17 cell differentiation mostly depends on the JNK and p38 pathways [391]. The reason for the discrepancies is unclear but could be due to the different mouse strains and experimental approaches used in these studies. Nonetheless, these findings suggest that the roles for r-Smad proteins in Th17 cell differentiation could be nuanced and complex. Multiple mechanisms are used by Smad proteins to control RORγt and IL-17 expression at both the protein and gene levels. The crosstalk between the TGF-β/r-Smad and MAPK signaling pathways is important for Treg and Th17 cell differentiation. TCR stimulation-induced MEKK2/MEKK3 and ERK activation leads to the phosphorylation of the linker regions of r-Smad2 and r-Smad3. Such phosphorylation suppressed the transactivation function of r-Smad2 and r-Smad3 in response to TGF-β stimulation [392]. Deletion of both MEKK2 and MEKK3 leads to an enhancement of TGF-β-promoted Treg and Th17 cell differentiation in vitro and in vivo [392]. Interestingly, MEKK2 and MEKK3 double knockout mice developed more severe EAE than wild-type mice, suggesting that MEKK2 and MEKK3 are preferentially required to dampen the TGF-β-controlled Th17 program [392]. The function of co-Smad4 in T cells was initially perplexing. In contrast to what was predicted for a protein that is central to TGFβR signaling, T-cell-specific depletion of co-Smad4 did not yield autoimmune symptoms or apparent T-cell activation as in T-cell-specific TGFβR knockout mice [250, 393]. Instead, T-cell-specific co-Smad4 deletion led to spontaneous development of cancer with increased Th17 cell differentiation [250, 393], suggesting that Smad4 has functions beyond promoting TGF-β signaling in T cells. A later study found that co-Smad4 depletion rescued lethal autoimmune disease in T-cell-specific TGFβRΙΙ-deficient mice [232], suggesting that co-Smad4 counterbalances TGFβR signaling. Indeed, closer examination revealed that co-Smad4-deficient T cells readily differentiated into Th17 cells in the presence of IL-6 and IL-21 in culture even when TGF-β signaling was abrogated, although TGF-β + IL-6-promoted Th17 cell differentiation was not obviously affected [250, 253, 394, 395]. In addition, during EAE development, although TGFβRII-deficient T cells fail to differentiate into Th17 cells, simultaneous knockout of co-Smad4 fully restores Th17 cell differentiation [253]. These findings suggest that co-Smad4 restrains Th17 cell differentiation in the absence of TGF-β stimulation. Without TGF-β, SKI is critical for the co-Smad4-mediated effects by associating with co-Smad4 on the *Rorc* locus but not the *Il17* locus and recruiting HDAC to the *Rorc* locus to restrain *Rorc* expression. Because SKI is very sensitive to TGF-β-induced protein degradation [396–398], low concentrations of TGF-β trigger SKI degradation and thus alleviate SKI/co-Smad4-complex-mediated suppression of *Rorc* expression [253]. Therefore, an important mechanism through which TGF-β promotes Th17 cell differentiation is to disrupt the SKI/co-Smad4 suppressive complex to allow *Rorc* expression and Th17 cell differentiation. Such a function of co-Smad4 appears to be context dependent. While co-Smad4 is indeed required to suppress Th17 cell differentiation under normal conditions, co-Smad4 is also required to promote pathogenic Th17 cell differentiation under febrile temperature [395]. High temperature promotes the heat shock response and the sumoylation of co-Smad4 and its nuclear translocation. Interestingly, febrile temperature induced co-Smad4 binding to the *Il17* loci [395]. These findings suggest that Smad proteins control Th17 cell differentiation in a context-dependent manner by integrating various environmental cues to positively and negatively regulate Th17 cell differentiation. Protein‒protein interactions have emerged as an important way to control Smad function. It would be of interest to further investigate how the interactomes of r-Smad2, r-Smad3, and co-Smad4 are dynamically regulated during Th17 cell differentiation under various conditions to identify critical factors for Th17 cell differentiation.

### Distinct roles for TGF-β superfamily members in regulating pathogenic and nonpathogenic functions of Th17 cells

Much effort has been devoted to understanding how TGF-β1 + IL-6 induces Th17 cells with low pathogenic function. Recently, we have seen increasing interest in understanding how pathogenic Th17 cell function is controlled due to its critical role in causing pathology [273, 274, 399–405]. TGF-β1 + IL-6-induced Th17 cells upregulate TGF-β3 in response to IL-23 to promote the pathogenic program, suggesting that TGF-β3 signaling can endow and enhance the pathogenic program of differentiating and differentiated Th17 cells [274]. However, whether TGF-β signaling is indeed involved in the de novo generation of pathogenic Th17 cells remains uncertain. In fact, a study suggests that TGF-β signaling is dispensable for the generation of pathogenic Th17 cells, particularly those differentiated by IL-6 + IL-1β + IL-23 [272]. Nonetheless, SKI degradation, a particularly sensitive readout for TGF-β signaling to allow Th17 cell differentiation [253], occurred under IL-6 + IL-1β + IL-23-polarizing conditions. Such SKI degradation was found to not be due to TGF-β signaling but rather to Activin A, a TGF-β superfamily member that is highly induced by IL-6 + IL-1β + IL-23. Activin A + IL-6 was shown to be sufficient to drive the differentiation of pathogenic Th17 cells that resemble IL-6 + IL-1β + IL-23-induced pathogenic Th17 cells. In addition, Activin A and its specific receptor I (ALK4) are critical for the generation of pathogenic Th17 cells to induce EAE. Furthermore, while TGF-β/ALK-5 signaling potently suppresses ERK activation, which is important for the pathogenic program of Th17 cells, Activin A/ALK4 signaling does not after ERK activation [406]. Therefore, different TGF-β superfamily members contribute to the generation and reinforcement of nonpathogenic and pathogenic Th17 cells through distinct and shared mechanisms. SKI suppresses the differentiation of both nonpathogenic and pathogenic Th17 cells. Ectopic SKI expression inhibits the generation of Th17 cells, especially pathogenic Th17 cells, both in culture and in vivo during EAE development [407]. SKI controls Th17 cell differentiation in a dose-dependent manner. Moderate stabilization of SKI in Arkadia-deficient T cells did not lead to substantial inhibition of Th17 cell differentiation [251], as certain levels of SKI expression can be tolerated for Th17 cell differentiation [406]. It is also possible that Arkadia knockout results in more complex effects, including the stabilization of the SnoN and Smad proteins and other uncharacterized targets that may affect co-Smad4 function, rather than only stabilizing the SKI protein [251].

### Localized TGF-β production for Th17 cell generation and function

While both TGF-β and Activin A can be produced by various cell types, T-cell-generated TGF-β and Activin A play nonredundant roles in Th17 cell function. Both TGF-β1 and Activin A produced by Th17 effector T cells are important for Th17 cells [239, 406]. In agreement, TGF-β1 is required for Th17 cell stability and for maintaining the nonpathogenic program. A study using IL-17-producing cell-specific TGFβ1 knockout and fate-mapping systems (*Tgfb1*^fl/fl^*Il17a*^Cre^*R26*^YFP^) revealed that autocrine TGFβ1 in Th17 cells maintains their stability by repressing the expression of IL-12Rβ2 and IL-27Rα [408]. TGF-β1-deficient Th17 cells tend to produce IFN-γ and become more pathogenic, exacerbating tissue inflammation in an adoptive EAE transfer model [408].

The aforementioned studies highlight the important functions of different TGF-β superfamily members and their signaling in controlling Th17 cell generation and function. However, whether Th17 cells can possibly be generated independent of TGF-β signaling remains incompletely answered. In mice doubly deficient in STAT6 and T-bet, Th17 cells are readily induced by IL-6 when TGFβRII signaling is attenuated by the expression of a dominant negative form of TGFβRII [409]. The purported mechanism was that failed Th1 and Th2 cell differentiation of STAT4/T-bet-double-knockout CD4^+^ T cells led to Th17 cell differentiation by default in the presence of IL-6. This posited mechanism is questionable because blocking Th1 and Th2 cell differentiation by other means does not result in Th17 cell differentiation by IL-6 stimulation alone. It would be interesting to assess whether Activin A-related signaling contributes to these observations. The abovementioned studies also highlight that Th17 cells are functionally heterogeneous with varying cytokine production profiles depending on the microenvironment in which they reside. Such high heterogeneity of Th17 cells could be due to different differentiation trajectories or functional plasticity, allowing dynamic adaptation to the changing environment. While TGF-β superfamily cytokine signaling is clearly instrumental in the generation and function of Th17 cells (Fig. 3B), the molecular underpinnings (especially the crosstalk with the pathways that sense environmental cues, including oxygen, metabolites, and chemicals) of TGF-β-controlled Th17 cell generation, maintenance, and function remain poorly defined and warrant further investigation.

The above discussion suggests that although Treg and Th17 cells are seemingly distinct cell types, their differentiation programs are related and share common pathways, especially the TGF-β pathway. In fact, conversion between Treg and Th17 cells can occur. An earlier study found that IL-6 abrogates Treg cell suppressive activity [386]. IL-6 was later shown to convert established Treg cells into Th17-like cells [250, 410]. The downregulation of Foxp3, which leads to the instability and plasticity of established Treg cells, is important to allow Treg-to-Th17 cell conversion because high levels of Foxp3 suppress RORγt to restrain Th17 cell differentiation [250, 279]. The attenuation and downregulation of Foxp3 expression are often associated with Th17 cell conversion of established Treg cells during immune pathologies [411], including type I diabetes, systemic autoimmunity, autoimmune arthritis, and neuroinflammation [149, 152, 412, 413]. Of note, while high levels of Foxp3 suppress Th17 cell differentiation [279], Foxp3 and IL-17 are not mutually exclusive, as cells expressing both can be found in vitro and in vivo [186, 250, 410]. How is TGF-β involved in Treg-Th17 conversion? Current evidence suggests that the strength of TGF-β signaling is important. High doses of TGF-β promote high levels of Foxp3 to favor Treg cell differentiation over Th17 cell differentiation even in the presence of IL-6 [253, 279]. In addition, while Activin-A promotes Th17 cell differentiation to promote inflammation [269], it does not promote Treg cell differentiation on its own [414], suggesting a potential role for Activin-A favoring Th17 cells over Treg cells. Therefore, it is reasonable to believe that strong TGF-β signaling will favor not only Treg cell generation but also their stability for immune homeostasis and suppression. Weak TGF-β and/or other TGF-β superfamily member signaling will permit Th17 cell differentiation and/or Treg-to-Th17 transdifferentiation under inflammatory conditions. What signaling molecules sense and interpret the strong vs. weak TGF-β signaling for Treg cell stability and Treg-Th17 cell transdifferentiation and how other TGF-β superfamily members are involved in Treg cell stability and Treg-Th17 cell transdifferentiation warrant further investigation to help understand the etiology and to develop treatments for immune diseases.

## Concluding remarks

The central canon for TGF-β’s function in Treg and Th17 cells is to balance the immune response in a context- and microenvironment-dependent manner through complex signaling pathways and molecular mechanisms. From an operational perspective, TGF-β signaling in Treg and Th17 cells must be intricate, sometimes nuanced, to support its ability to integrate and respond to a plethora of environmental cues through dose- and context-dependent mechanisms. This ability of TGF-β signaling allows T cells to correctly interpret cellular and molecular contexts and mount defined, precise responses in a malleable way to adapt to the ever-changing microenvironment. TGF-β accomplishes these daunting tasks by wiring and rewiring cell-intrinsic pathways involving different cellular factors in varying combinations. When studying TGF-β signaling and response, cellular and molecular contexts matter. Future research efforts should focus on revealing how TGF-β signaling components control T-cell responses in cell-type- and niche-specific contexts that are meaningful for immunity and diseases.

To fully appreciate the intricate, context-dependent roles of TGF-β signaling in T-cell function and to understand some of the seemingly conflicting observations, it is important to consider the following: First, the source of TGF-βs can be diverse and redundant, as TGF-βs with similar biochemical properties can be produced by broad cell types in secreted or membrane-bound forms. Therefore, the cell types involved and their physical position relative to Treg and Th17 cells in a specific niche should be considered. Second, to signal, mature TGF-β needs to be freed from LAP, a process that involves complex regulation. Activating TGF-β involves various mechanisms, including acidification, proteases, plasmin, matrix metalloproteases, thrombospondin-1, and integrins [415, 416]. Conditions, such as inflammation, that mobilize these mechanisms will impact the availability of active TGF-β and influence the reliance and responsiveness of T cells to TGF-β to achieve balanced responses. Third, under complex conditions in the microenvironment in vivo, other TGF-β superfamily members may also be involved in TGF-β signaling in Treg and Th17 cells in a context-dependent manner. TGF-β superfamily members can cooperate through shared or unique components and pathways to mitigate or exacerbate some of the effects observed under various settings. Finally, due to broad crosstalk between TGF-β signaling pathways and other signaling pathways, transcription factors, and epigenetic regulators, the “molecular contexts” in a cell will likely substantially affect the signaling and functional outputs of TGF-β stimulation. Therefore, it is important to comprehensively understand these “molecular contexts” using multiomics approaches, ideally at the single-cell level, to fully appreciate how intricately TGF-β signaling functions in a context-dependent fashion.
